# Diagnosis of thyroid nodules on ultrasonography by a deep convolutional neural network

**DOI:** 10.1038/s41598-020-72270-6

**Published:** 2020-09-17

**Authors:** Jieun Koh, Eunjung Lee, Kyunghwa Han, Eun-Kyung Kim, Eun Ju Son, Yu-Mee Sohn, Mirinae Seo, Mi-ri Kwon, Jung Hyun Yoon, Jin Hwa Lee, Young Mi Park, Sungwon Kim, Jung Hee Shin, Jin Young Kwak

**Affiliations:** 1 Department of Radiology, CHA Bundang Medical Center, CHA University, Seongnam, Republic of Korea; 2 Department of Computational Science and Engineering, Yonsei University, Seoul, Republic of Korea; 3 Department of Radiology, Severance Hospital, Research Institute of Radiological Science, Yonsei University, College of Medicine, 50 Yonsei-ro, Seodaemun-gu, Seoul, 03722 Republic of Korea; 4 Department of Radiology, Gangnam Severance Hospital, Yonsei University, College of Medicine, Seoul, Republic of Korea; 5 Department of Radiology, Kyung Hee University Hospital, College of Medicine, Kyung Hee University, Seoul, Republic of Korea; 6 Department of Radiology and Center for Imaging Science, Thyroid Center, Samsung Medical Center, Sungkyunkwan University School of Medicine, 81, Irwon-ro, Gangnam-gu, Seoul, 06351 Republic of Korea; 7 Department of Radiology, Dong-A University Hospital, Dong-A University, College of Medicine, Busan, Republic of Korea; 8 Department of Diagnostic Radiology, Busan Paik Hospital, Inje University, College of Medicine, Busan, Republic of Korea

**Keywords:** Cancer, Computational biology and bioinformatics, Endocrinology

## Abstract

The purpose of this study was to evaluate and compare the diagnostic performances of the deep convolutional neural network (CNN) and expert radiologists for differentiating thyroid nodules on ultrasonography (US), and to validate the results in multicenter data sets. This multicenter retrospective study collected 15,375 US images of thyroid nodules for algorithm development (n = 13,560, Severance Hospital, SH training set), the internal test (n = 634, SH test set), and the external test (n = 781, Samsung Medical Center, SMC set; n = 200, CHA Bundang Medical Center, CBMC set; n = 200, Kyung Hee University Hospital, KUH set). Two individual CNNs and two classification ensembles (CNNE1 and CNNE2) were tested to differentiate malignant and benign thyroid nodules. CNNs demonstrated high area under the curves (AUCs) to diagnose malignant thyroid nodules (0.898–0.937 for the internal test set and 0.821–0.885 for the external test sets). AUC was significantly higher for CNNE2 than radiologists in the SH test set (0.932 vs. 0.840, *P* < 0.001). AUC was not significantly different between CNNE2 and radiologists in the external test sets (*P* = 0.113, 0.126, and 0.690). CNN showed diagnostic performances comparable to expert radiologists for differentiating thyroid nodules on US in both the internal and external test sets**.**

## Introduction

Thyroid nodules are a very common clinical problem and the prevalence of thyroid nodules is 19–68% with the use of high-resolution ultrasonography (US)^1^. Differentiating malignancy is an important clinical process, as malignancy occurs in 7–15% of detected thyroid nodules depending on various risk factors^2^. The worldwide incidence of thyroid cancer is 567,000 cases and incidence has steadily increased to this number since the early 1980s largely due to improvements in detection and diagnosis^3^.

US is the most sensitive and core modality for diagnosing thyroid nodules^4^. Radiologists use several US features to differentiate malignancy and benignity in thyroid nodules, such as hypoechogenicity, solidity, microcalcifications, taller-than-wide shape, and not circumscribed margin^4–6^. However, US does not show a high accuracy for differentiation, and its diagnostic value varies from study to study^7,8^. Moreover, US is dependent on the performer and interobserver variability exists when assessing thyroid nodules according to US features^8^.

There have been several attempts to diagnose thyroid nodules with artificial intelligence, the first being machine learning, but machine learning either underperformed^9^ or showed similar accuracy to radiologists^10^. Machine learning relies on predefined engineered features extracted by expert radiologists, and therefore, the extraction can be arbitrary and important features can either be omitted or redundantly included^11^. In deep learning with the convolutional neural network (CNN), feature extraction belongs to the training process. Thus, the network chooses features and makes decisions^11^. Owing to performance improvements in computer processing, deep learning technology is evolving rapidly in the field of medical imaging with success^11,12^. Deep learning based on big data has shown outstanding results in image classification^13–15^. A few studies have used deep learning to diagnose thyroid cancers on US, and according to previous studies, deep learning can differentiate benign and malignant thyroid nodules with similar diagnostic performances to radiologists^16–19^.

In this study, we evaluated and compared the diagnostic performance of deep CNN for differentiating thyroid nodules on US with expert radiologists, and tried to further validate our results with multicenter data sets.

## Methods

### Study cohorts

This multicenter retrospective study was conducted at four tertiary referral institutions of South Korea (Severance Hospital, Samsung Medical Center, CHA Bundang Medical Center, and Kyung Hee University Hospital) with study cohorts made up of patients who visited each institution between 2004 and 2019. The study cohorts were screened for thyroid nodules 10 mm or larger with appropriate cytologic or pathologic reports. Approval was obtained from the institutional review boards (IRBs) of all institutions, and requirement for informed consent was waived as the study design was based on a retrospective review of medical records and radiologic images (IRBs, Severance Hospital: 4-2019-0163, Samsung Medical Center: 2019-04-092-001, CHA Bundang Medical Center: 2019-01-042-002, and Kyung Hee University Hospital: 2019-05-009). All experiments were performed in accordance with relevant guidelines and regulations. All images and pathologic data were anonymized before being transferred between different hospitals.

**Figure 1:**
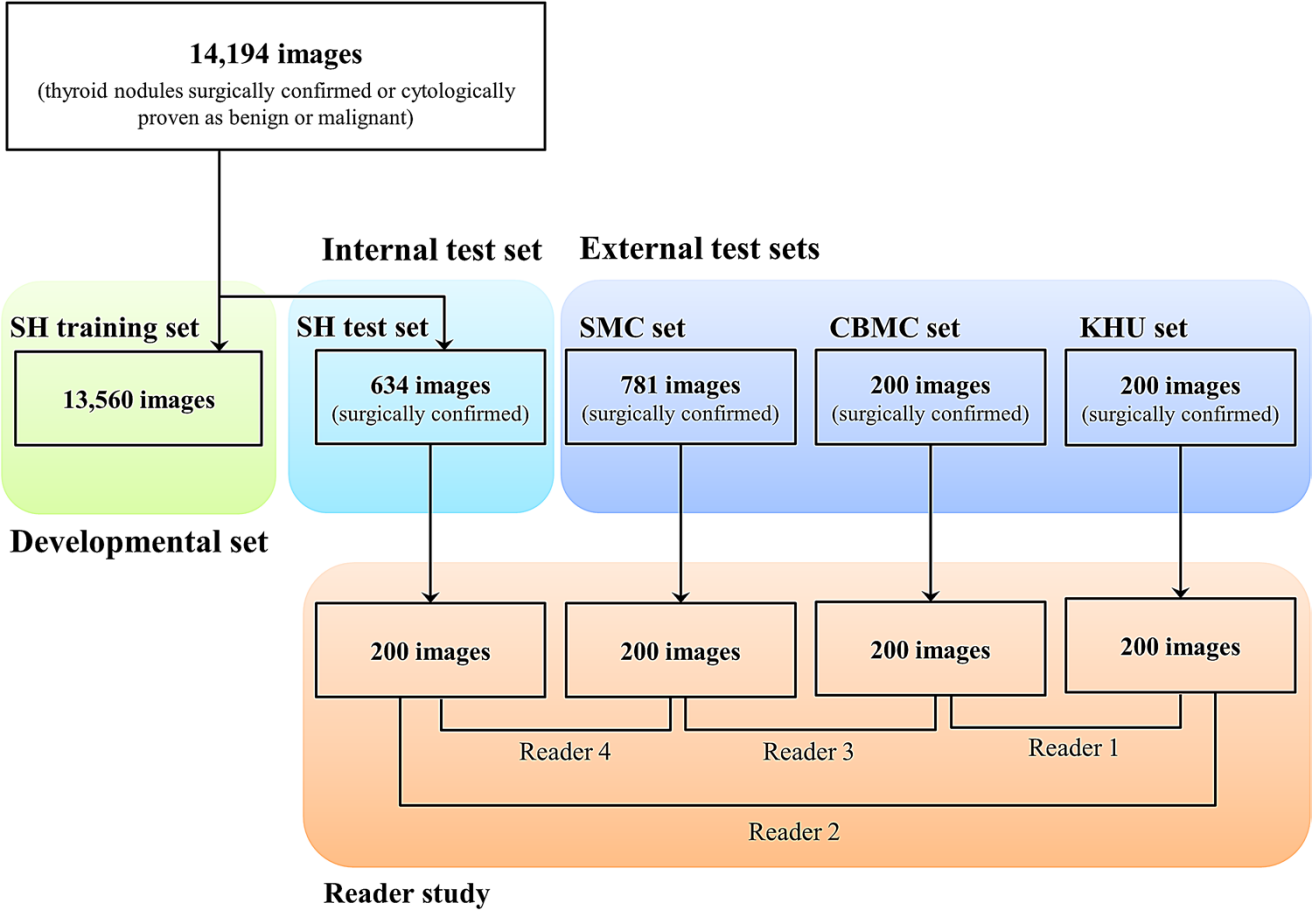
Diagram of the study cohort. For the algorithm development, 13,560 images of thyroid nodules were collected from Severance Hospital (SH training set). For the internal test, 634 images of thyroid nodules were additionally obtained from Severance Hospital (SH test set). For the external test, 1,181 images of thyroid nodules were obtained from three different hospitals (Samsung Medical Center, SMC set; CHA Bundang Medical Center, CBMC set; Kyung Hee University Hospital, KUH set). For the four test sets, 200 images were selected and four readers retrospectively reviewed two sets of images to compare diagnostic performance between expert radiologists and CNN.

To develop the algorithm, 13,560 images of thyroid nodules which were either surgically confirmed or cytologically proven as benign (category II) or malignant (category VI) on the Bethesda system were collected from one institution (Severance Hospital, SH training set) (Fig. 1). Thyroid nodules diagnosed as category I, III, IV, or V without further confirmation were not included. One of 11 US machines was used (Supplementary method). For the internal test, 634 images of surgically confirmed thyroid nodules were obtained, and these images were not included in the development of the algorithm (SH test set). For the external test, 1,181 additional images of surgically confirmed thyroid nodules were obtained from three different hospitals (781 images from Samsung Medical Center, SMC set; 200 from CHA Bundang Medical Center, CBMC set; 200 from Kyung Hee University Hospital, KUH set). All US images of each patient were reviewed and a representative image of each thyroid nodule was selected and saved as a JPEG file in the picture archiving and communication system. Using the Microsoft Paint program (version 6.1; Microsoft Corporation, Redmond, WA, USA), square region-of-interests (ROIs) were drawn on nodules of the SH training and SH test sets to cover the whole nodule by one of four expert radiologists (J.H.Y., J.H.L., Y.M.P., and J.Y.K.). For the SMC set, CBMC set, and KUH set, ROIs were drawn on nodules by affiliated expert radiologists who collected the images.

### Image acquisition

**Figure 2:**
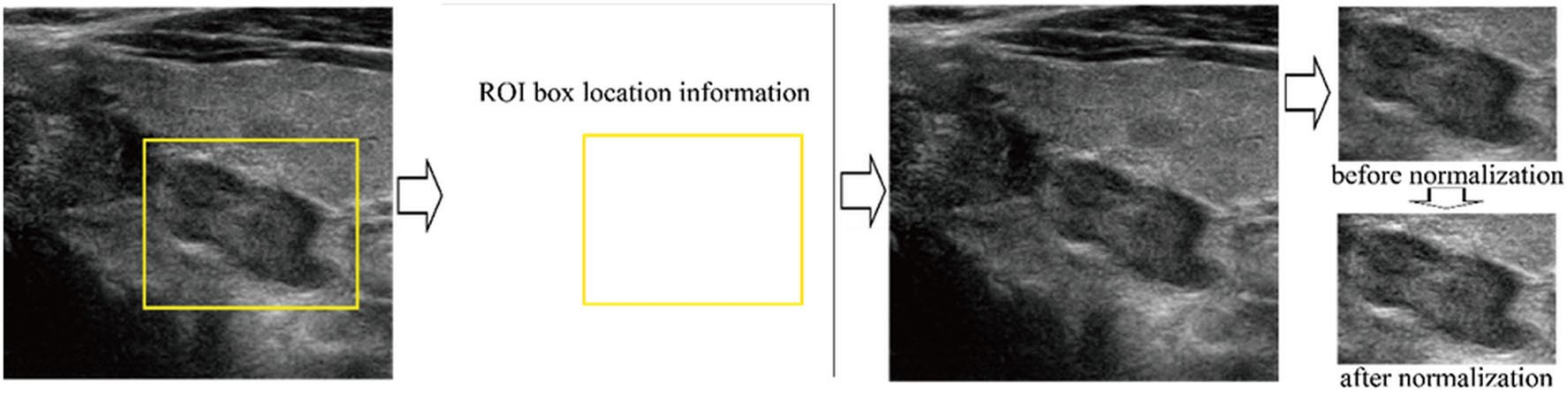
Image acquisition process. To extract the ROI without unnecessary interference from the color bounding box used to indicate the ROI’s border, location information was harvested and applied to a duplicate image that did not have the ROI box drawn on it.

In the deep learning process, only the ROI was used as input data to increase algorithm performance by discarding irrelevant information. To extract the ROI without unnecessary interference from the color bounding box used to indicate the border of the ROI, location information was harvested and applied to a duplicate image that did not have the ROI box as shown in Fig. 2. The total number of training data was 13,560 that included 7,160 malignant and 6,400 benign nodule images. In order to balance the number of the two categories, 760 benign nodule images were randomly chosen and left–right mirroring images were generated. Hence, a total of 14,320 images was used in training. As another important pre-processing step, all the images were first normalized so that the pixel values of the grayscale images lied within the range of 0 and 255 to induce similar data distribution and faster convergence of the deep learning algorithm. Then, image sizes were adjusted to fit the structure of each deep learning algorithm using simple linear interpolation between pixels.

### Deep CNN implementation

**Figure 3:**
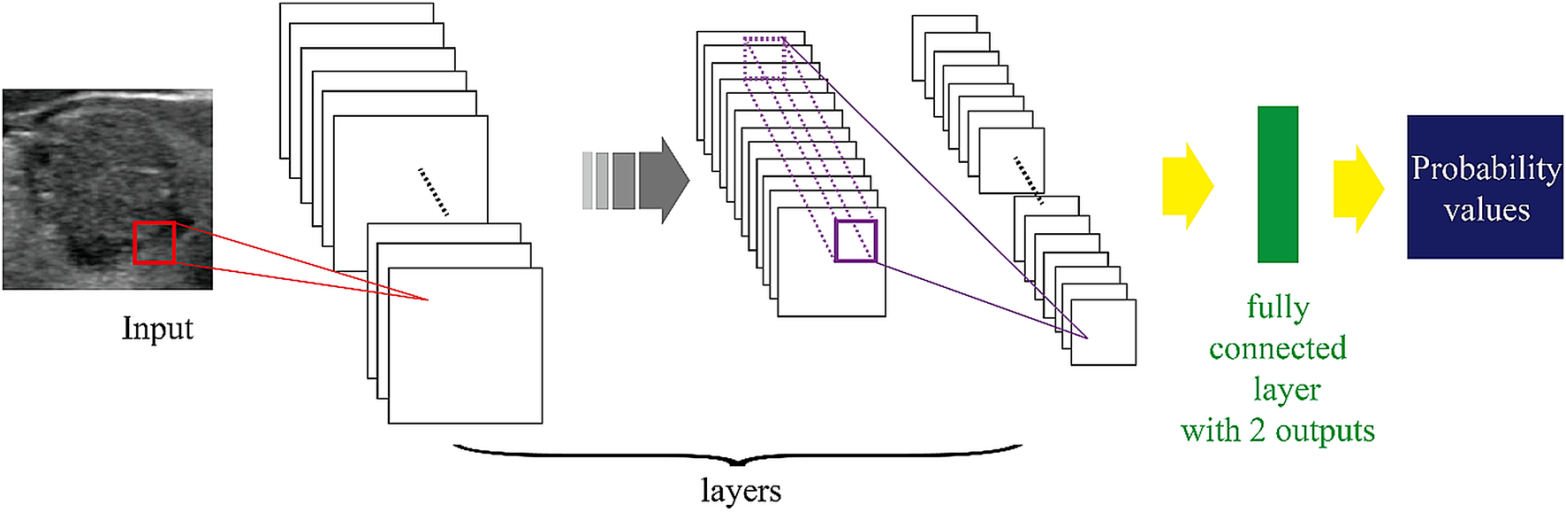
Structure of CNN with fine-tuning. The last few layers are modified to produce two output results.

In general, a deep learning process requires big data which is hard to obtain with current medical imaging. For this reason, we utilized transfer learning that adopts a CNN pre-trained with a huge amount of non-medical images and applied it to the included cohorts^20,21^. The 10 CNNs used in this study were AlexNet^22^, SqueezeNet^23^, VGG16^24^, VGG19^25^, GoogLeNet^26^, ResNet18^27^, ResNet50^27^, Inception-v3^28^, DenseNet-201^29^, and InceptionResNetV2^30^ which were all pre-trained with a total of 1,281,167 non-medical images. Each CNN was fine-tuned with 14,320 images from the SH training set and was tested with internal and external test sets. A diagram detailing CNN implementation is shown in Fig. 3.

**Figure 4:**
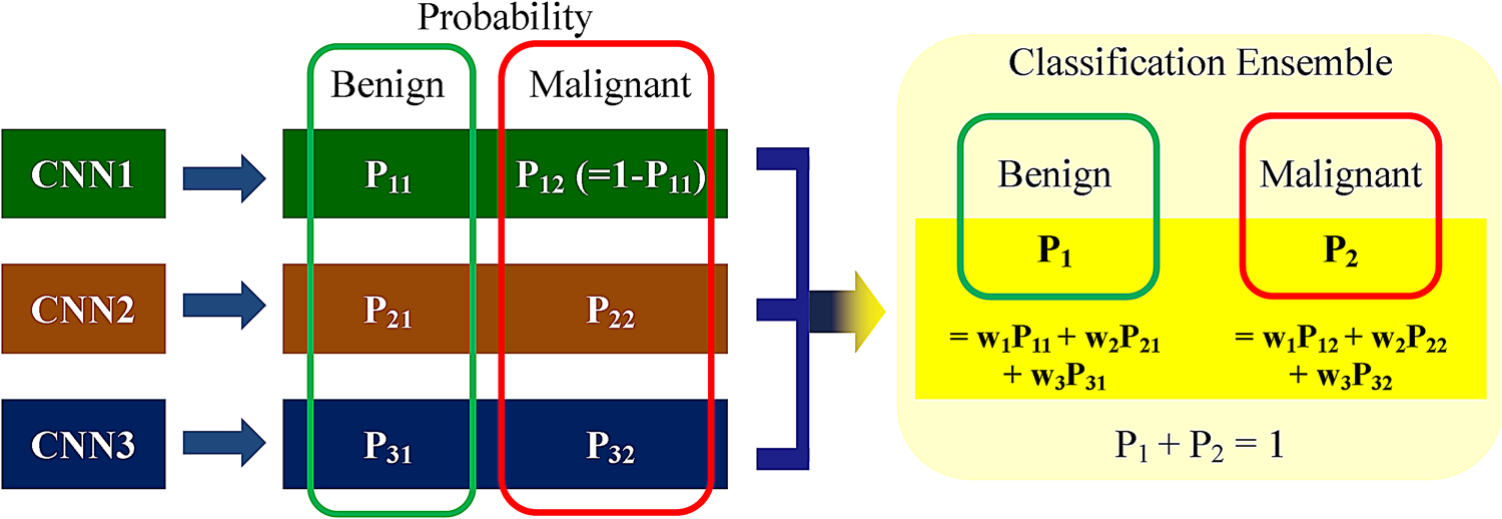
Structure of the classification ensemble. When multiple CNNs were selected, the probability results were collected from each CNN as shown in Fig. 3 and these probabilities were averaged to generate a new probability for the final decision.

Each CNN made slightly different decisions because discriminative features were developed from results that were dependent on how the layers of each CNN were designed. Therefore, to gather the majority opinion, we used a classification ensemble that combined the results of each CNN after the training process as shown in Fig. 4. The classification ensemble selected multiple CNNs and collected the probabilities of the selected CNNs. These probabilities were weighted with the AUC obtained from the validation process, in which 10% of the training data was randomly chosen and used for validation, and then the sum was used in decision making. During this process, 1,013 possible combinations were analyzed.

Based on the performance of individual CNN results and classification ensemble results with validation data, we chose four CNNs including two individual CNNs, ResNet50 (CNN1) and InceptionResNetV2 (CNN2), and two classification ensembles, AlexNet-GoogLeNet-SqueezeNet ensemble (CNNE1) and AlexNet-GoogLeNet-SqueezeNet-InceptionResNetv2 ensemble (CNNE2).

### CNN evaluation and statistical analysis

CNN1, CNN2, CNNE1, and CNNE2 were tested with the internal test set (634 images) and three external test sets (1,181 images) and results were presented with the cancer probability. The gold standard was the pathologic report. From the SH test and SMC set, 200 images were randomly selected from each hospital and all 200 images of the CBMC and KHU set were used to compare the diagnostic performances of the expert radiologists and CNN. Four radiologists who were experts in thyroid imaging (16–23 years of experience) reviewed images and evaluated nodules according to the American College of Radiology (ACR) Thyroid Imaging, Reporting and Data System (TI-RADS) (TR1-TR5)^6^. The readers reviewed two sets of images (400 images) obtained at hospitals other than their affiliated hospitals and were blinded to pathologic results (Fig. 1). We calculated the sensitivity, specificity, positive predictive value (PPV), negative predictive value (NPV), and accuracy of the CNNs and readers for predicting malignancy. Receiver-operating-characteristic (ROC) analysis was performed and the area under the curve (AUC) was also calculated. F1 metrics were also calculated. To compare diagnostic performances among the four CNNs and between the CNNs and expert radiologists, logistic regression with the generalized estimating equation was used and AUC was compared with multireader-multicase ROC analysis with *RJafroc* (version 1.2.0). Youden’s index was used to set the cut-off values for sensitivity, specificity, PPV, NPV, and accuracy. We divided all nodules of the test sets into 5 categories according to the cancer probabilities presented by CNNE2 based on the ACR TI-RADS, which were cancer risk levels of no more than 2% for TR1 and TR2 nodules, 5% for TR3 nodules, 5% to 20% for TR4 nodules, and at least 20% for TR5 nodules^6^. Interobserver agreements between two readers were evaluated using weighted Cohen’s kappa statistics with the *irr* package (version 0.84.1). Statistical analyses were performed with R Statistical Software (version 3.6.0.; R Foundation for Statistical Computing, Vienna, Austria) and SAS (version 9.4, SAS Institute Inc., Cary, NC, USA). Two-sided* P* < 0.05 was considered to have statistical significance.

## Results

The baseline characteristics of the study cohorts are listed in Table 1. CNNs demonstrated high AUCs for differentiating malignant thyroid nodules which were 0.898–0.937 for the SH test set, 0.854–0.881 for the SMC set, 0.854–0.885 for the CBMC set, and 0.821–0.854 for the KUH set (Supplementary Table S1). For the SH test set, CNNE1 and CNNE2 showed higher AUCs compared to CNN1 or CNN2 (Supplementary Fig. S1). We chose CNNE2 to compare diagnostic performances between CNN and expert radiologists. Detailed diagnostic performances of CNN1, CNN2, CNNE1, and CNNE2 are shown in Supplementary Table S1. To calculate the diagnostic performance of each cohort and to compare them with the diagnostic performances of the expert radiologists, the cut-off value of cancer probability was calculated from the SH test set according to Youden’s index (0.6 for CNN1, 0.5 for CNN2, 0.6 for CNNE1, and 0.6 for CNNE2).

**Table 1:** Baseline characteristics of the study cohorts.

	SH training set (n = 13,560)	SH test set (n = 634)	SMC set (n = 781)	CBMC set (n = 200)	KUH set (n = 200)
Age, mean ± SD (years)	47.4 ± 13.7	44.6 ± 13.0	47.2 ± 12.9	48.7 ± 13.0	49.6 ± 13.9
Size, mean ± SD (mm)	20.3 ± 11.4	19.6 ± 12.3	23.6 ± 13.4	21.2 ± 11.5	22.4 ± 11.3
**Sex**					
Female	10,675 (78.7%)	484 (76.3%)	571 (73.1%)	161 (80.5%)	151 (75.5%)
Male	2,885 (21.3%)	150 (23.7%)	210 (26.9%)	39 (19.5%)	49 (24.5%)
**Cytopathologic results**					
Malignancy	7,160 (52.8%)	539 (85.0%)	538 (68.9%)	118 (59.0%)	98 (49.0%)
Benign	6,400 (47.2%)	95 (15.0%)	243 (31.1%)	82 (41.0%)	102 (51.0%)
**Cancer subtype**^**a**^					
Papillary cancer	6,478 (96.5%)	519 (96.3%)	405 (75.3%)	116 (98.3%)	97 (99.0%)
Follicular cancer	148 (2.2%)	10 (1.9%)	126 (23.4%)	0	0
Medullary cancer	30 (0.4%)	6 (1.1%)	3 (0.6%)	1 (0.8%)	0
Anaplastic cancer	20 (0.3%)	0	1 (0.2%)	1 (0.8%)	1 (1.0%)
Other	36 (0.5%)	4 (0.7%)	3 (0.6%)	0	0

^a^Cancer subtype is listed only for surgically confirmed cases.

TR4 was used as the cut-off value to evaluate the diagnostic performance of expert radiologists. AUC of expert radiologists to diagnose thyroid nodules was 0.842 and 0.838 for the SH test set, 0.799 and 0.847 for the SMC set, 0.850 and 0.810 for the CBMC set, and 0.842 and 0.897 for the KUH set (Table 2). Sensitivity was 89.2% and 94.0% for the SH test set, 93.7% and 88.7% for the SMC set, 89.0% and 90.7% for the CBMC set, and 91.8% for the KUH set. Specificity was 67.7% and 50.0% for the SH test set, 39.7% and 56.9% for the SMC set, 67.1% and 45.1% for the CBMC set, and 60.8% and 71.6% for the KUH set. Interobserver agreements between radiologists to diagnose thyroid nodules were moderate to good (0.573–0.657).

**Table 2:** Diagnostic performances of expert radiologists and CNNE2.

	SH test set (n = 200)				SMC set (n = 200)				CBMC set (n = 200)				KUH set (n = 200)			
	Reader 2	Reader 3	Average^b^	CNNE2	Reader 3	Reader 4	Average	CNNE2	Reader 1	Reader 4	Average	CNNE2	Reader 1	Reader 2	Average	CNNE2
Sensitivity (%)^a^	89.2 (83.5–93.1)	94.0 (89.2–96.7)	91.6 (87.3–94.5)	83.7 (77.3–88.6)	93.7 (88.3–96.7)	88.7 (82.4–93.0)	91.2 (86.4–94.4)	78.2 (70.6–84.2)	89.0 (82.0–93.5)	90.7 (84.0–94.8)	89.8 (84.4–93.5)	94.1 (88.1–97.2)	91.8 (84.5–95.9)	91.8 (84.5–95.9)	91.8 (85.5–95.5)	91.8 (84.5–95.9)
Specificity (%)^a^	67.7 (50.5–81.1)	50 (33.8–66.2)	58.8 (44.1–72.1)	91.2 (76.0–97.1)	39.7 (28.0–52.7)	56.9 (44.0–68.9)	48.3 (37.1–59.6)	93.1 (83.0–97.4)	67.1 (56.2–76.4)	45.1 (34.7–56.0)	56.1 (46.5–65.2)	62.2 (51.3–72.0)	60.8 (51.0–69.8)	71.6 (62.1–79.5)	66.2 (57.7–73.7)	59.8 (50.0–68.9)
Accuracy (%)^a^	85.5 (79.9–89.7 )	86.5 (81.0–90.6- )	86.0 (81.3–89.7)	85.0 (79.4–89.3)	78.0 (71.7–83.2)	79.5 (73.3–84.5)	78.8 (73.2–83.4)	82.5 (76.6–87.2 )	80.0 (73.9–85.0)	72.0 (65.4–77.8)	76.0 (70.4–80.8)	81.0 (75.0–85.9)	76.0 (69.6–81.4)	81.5 (75.5–86.3)	78.8 (73.3–83.4)	75.5 (69.1–81.0)
PPV (%)^a^	93.1 (87.9–96.1)	90.2 (84.8–93.8)	91.6 (86.7–94.8)	97.9 (93.7–99.3)	79.2 (72.4–84.7)	83.4 (76.6–88.6)	81.2 (74.7–86.3)	96.5 (91.1–98.7)	79.6 (71.8–85.6)	70.4 (62.7–77.1)	74.7 (67.3–80.8)	78.2 (70.6–84.2)	69.2 (60.8–76.6)	75.6 (67.1–82.5)	72.3 (64.3–79.1)	68.7 (60.3–76.1)
NPV (%)^a^	56.1 (40.8–70.3)	63.0 (43.8–78.8)	58.8 (43.8–72.4)	53.5 (40.7–65.8)	71.9 (54.2–84.7)	67.4 (53.2–78.9)	69.1 (55.4–80.2)	63.5 (52.8–73.0)	80.9 (69.8–88.6)	77.1 (63.2–86.8)	79.3 (68.9–86.9)	87.9 (76.8–94.1)	88.6 (78.8–94.2)	90.1 (81.5–95.0)	89.4 (81.3–94.3)	88.4 (78.5–94.1)
AUC	0.842 (0.771–0.914)	0.838 (0.762–0.913)	0.840 (0.806–0.873)	0.932 (0.885–0.978)	0.799 (0.734–0.863)	0.847 (0.793–0.901)	0.823 (0.706–0.940)	0.899 (0.858–0.940)	0.850 (0.798–0.902)	0.810 (0.754–0.866)	0.830 (0.752–0.909)	0.885 (0.839–0.930)	0.842 (0.79–0.894)	0.897 (0.855–0.94)	0.870 (0.752–0.987)	0.854 (0.800–0.908)
F1	91.1	92.0	91.6	90.3	85.8	86.0	85.9	86.4	84.0	79.3	81.5	85.4	79.0	83.0	80.9	78.6

^a^To calculate the diagnostic performances of each cohort, a cut-off value of 0.6 for cancer probability was used for CNNE2 and ACR TI-RADS category 4 was used for readers.

^b^The average reader performance was calculated.

**Figure 5:**
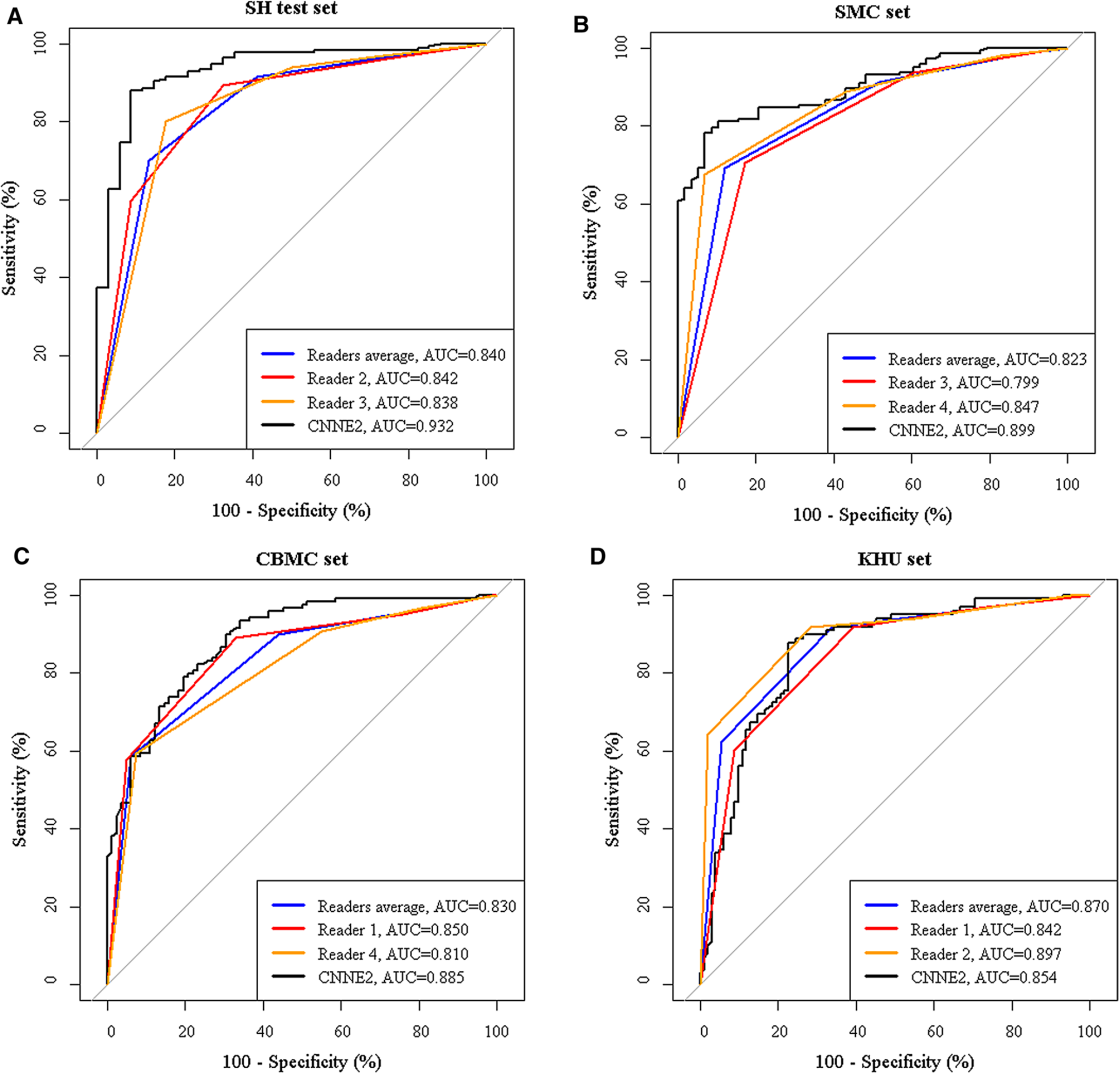
ROC curves of CNNE2 and expert radiologists for differentiating thyroid nodules. A. AUC of CNNE2 was significantly higher than radiologists in the SH test set (0.932 vs. 0.840, *P* < 0.001). AUC of CNNE2 was higher than radiologists in the SMC set (B) and CBMC set (C) without statistical significance (0.899 vs. 0.823 and 0.885 vs. 0.830, *P* = 0.113 and 0.126) D. AUC of radiologists was higher than CNNE2 in the KHU set without statistical significance (0.870 vs. 0.854, *P* = 0.690). (Black: CNNE2, Blue: readers average, red and orange: individual reader).

The ROC curves of readers and CNNE2 are demonstrated in Fig. 5. We compared the average diagnostic performances of radiologists to those of CNNE2. AUC of CNNE2 was significantly higher than that of radiologists in the SH test set (0.932 vs. 0.840, *P* < 0.001). AUC of CNNE2 was also higher than those of radiologists in the SMC set and CBMC set (0.899 vs. 0.823 and 0.885 vs. 0.830) while AUC of radiologists was higher than that of CNNE2 in the KHU set (0.870 vs. 0.854), but these results were not statistically significant (*P* = 0.113, 0.126, and 0.690, respectively). The expert radiologists showed significantly higher sensitivity than CNNE2 in the SH test set and SMC set (91.6% vs. 83.7% and 91.2% vs. 78.2%, *P* < 0.002). CNNE2 showed significantly higher specificity than radiologists in the SH test set and SMC set (91.2% vs. 58.8% and 93.1% vs. 48.3%, *P* < 0.001). CNNE2 also showed significantly higher PPV than radiologists in the SH test set and SMC set (97.9% vs. 91.6% and 96.5% vs. 81.2%, *P* < 0.001) and higher NPV than radiologists in the CBMC set (87.9% vs. 79.3%, *P* = 0.038). Other diagnostic performances were not statistically different between radiologists and CNNE2.

When we divided the 1,815 nodules of the test sets into 5 categories using cancer probabilities of CNNE2 according to the recommended cancer risk of the ACR TI-RADS categories, the malignancy rate was 0% for TR2, 11.1% for TR3, 18.8% for TR4, and 77.2% for TR5 (Supplementary Table S2). These results were concordant within the range of cancer risk suggested by ACR TI-RADS except for TR3, which was higher than 2–5%.

## Discussion

CNN is used in medical imaging for detection, classification, and segmentation tasks^12^. In our study, we used CNNs for classification tasks with supervised training to differentiate thyroid nodules on US. Our study was based on a large number of images and its results were externally validated at three different hospitals. We compared the performances of CNNs and four expert radiologists and found CNNs to show higher or comparable diagnostic performances to radiologists in the diagnosis of thyroid nodules on US. Similar results were found in both the internal and external test sets.

We tested 10 CNNs and 1,013 ensemble CNNs to select the best performing CNN for thyroid nodule differentiation. We selected 2 CNNs (CNN1 and CNN2) and two ensembles (CNNE1 and CNNE2). Ensemble CNNs showed higher AUCs than CNNs when diagnosing thyroid nodules on US. Previous studies on diagnosing thyroid nodules also used ensemble CNNs because ensemble CNNs showed better performance than CNNs^16,17,31^. In our study, CNNE2 showed a high AUC for the differentiation of thyroid nodules which was 0.932 for the SH test set, 0.899 for the SMC set, 0.885 for the CBMC set, and 0.854 for the KHU set. The AUC of our study was comparable with other previous studies which demonstrated AUCs of 0.893–0.947^16,17,31^.

Expert radiologists in our study used ACR TI-RADS to score the thyroid nodules, and the AUCs of radiologists ranged from 0.799 to 0.897. We used TR4 as the cut-off value when calculating sensitivity, specificity, PPV, NPV, and accuracy according to Youden’s index and this was different from a previous study which used TR5 to evaluate radiologists’ performances^16^. However, the AUC of expert radiologists was not given in this previous study, so we could not compare AUCs. Although a different cut-off value was used, the sensitivity of our study ranged 88.7–94.0% and the specificity ranged 39.7–71.6% which was similar to the previous study which showed a sensitivity of 85.6–98.4% and a specificity of 51.2–78.8%^16^. The accuracy of radiologists was 72.0–86.5% in our study, which was again similar to the previous study which showed an accuracy of 70.8–81.8%^16^. In this study, interobserver agreement between expert radiologists to diagnose thyroid nodules was moderate to good. Therefore, we found it hard to sustain objectivity and reproducibility even with expert radiologists performing the US. Furthermore, inexperienced radiologists showed even lower interobserver agreements than expert radiologists in previous studies when diagnosing thyroid nodules on US^8,32^. CNN has the advantage of producing both objective and reproducible results.

When we compared the average diagnostic performances of expert radiologists and CNNE2, CNNE2 demonstrated comparable or better performance than radiologists. AUC of CNNE2 was significantly higher (0.932) than radiologists (0.840) in the internal set. In the external test sets, AUC of CNNE2 was higher than that of radiologists without statistical significance. CNNE2 showed higher sensitivity, specificity, PPV, and NPV than radiologists in several subsets. In the previous study, the CNN also reported higher or similar diagnostic performances over radiologists^16–19^.

We divided all nodules into 5 categories according to the cancer probabilities presented by CNNE2 in the test sets based on the cancer risks suggested by the ACR TI-RADS categories^6^. We found that the malignancy rate was 0% for TR2, 11.1% for TR3, 18.8% for TR4, and 77.2% for TR5. These results were within the range of cancer risk suggested by ACR TI-RADS except for TR3, which was higher than 2–5%. The higher cancer risk might be due to the very small overall number of nodules classified as TR3 in our study. CNNE2 demonstrated cancer probabilities similar with ACR TI-RADS, and this implies that CNNs can play a role in risk stratification as well as disease diagnosis. To the best of our knowledge, our study is the first to attempt to use the CNN for risk stratification.

We acknowledge that there are several limitations in this study. First, we only trained and tested a representative single image of each nodule because we could not obtain volume data from US unlike computed tomography or magnetic resonance imaging. Selecting a representative image was itself influenced by the experience and skills of the performing clinician. It is evident that the deep learning process still requires a significant amount of human intervention when images are selected. In real practice, physicians usually evaluate thyroid nodules using real-time US information, not a representative image. In the near future, we need to study the real-time automated application of CNN or CNN with improved technology allowing the collection of US volume data. Second, although we validated the results externally in three different hospitals and the chosen CNN also performed well, the study population was enrolled from one nation, and further multi-national center studies are needed to validate the results. Third, we performed this study in tertiary centers equipped with high-quality US machines. Therefore, it might be difficult to produce desirable performances when CNN is applied to images of relatively lower quality obtained at outside clinics. Fourth, we used Youden’s index to set the cut-off value for TI-RADS and the performances of expert radiologists would vary with different cut-off scores for TI-RADS. Fifth, the prevalence of malignancy differed among the four hospitals and this might have influenced the diagnostic performances^33^. Because all cases of the test sets were surgically confirmed cases, the malignancy proportions of these sets were higher than what would be seen in everyday practice. However, controlling disease prevalence evenly across cohorts is difficult because the prevalence of malignancy differs between individual hospitals as institutional size itself is different. Sixth, we included nodules surgically confirmed or cytologically proven as benign or malignant in the training set. False-positive or false-negative cases might exist for the cytologically proven nodules, but according to the malignancy rates reported by Bethesda system for category II and VI, the number of these cases would be too small to affect the training process.

In conclusion, CNN showed comparable diagnostic performances to expert radiologists in differentiating thyroid nodules on US in both the internal and external test sets.

## Supplementary information

Supplementary Figure S1a.

Supplementary Figure S1b.

Supplementary Figure S1c.

Supplementary Figure S1d.

Supplementary information.

## Data Availability

No datasets were generated or analysed during the current study.
