# Supplementary figures and images for: Diagnosis of thyroid nodules on ultrasonography by a deep convolutional neural network
Source: Sci Rep. 2020 Sep 17;10:15245. doi: 10.1038/s41598-020-72270-6 (PMC7498581; doi:10.1038/s41598-020-72270-6)

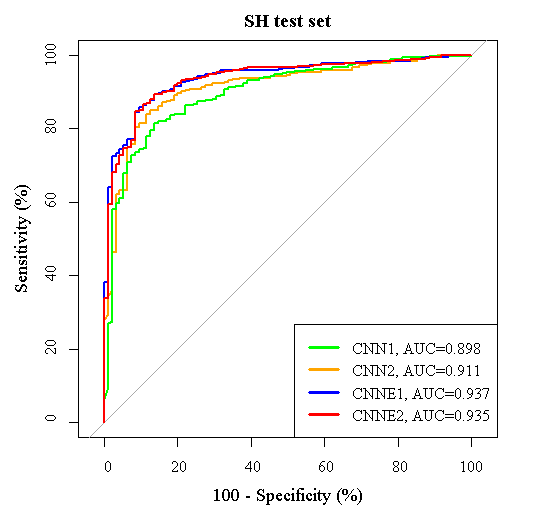

Supplement: Supplementary file 1 — Supplementary Figure S1a. [file 41598_2020_72270_MOESM1_ESM.tiff]

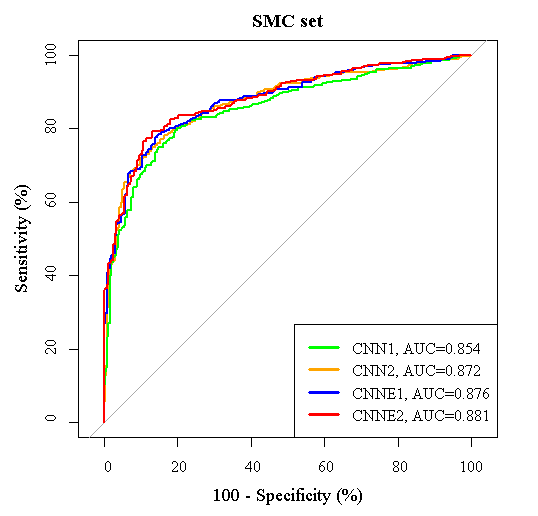

Supplement: Supplementary file 2 — Supplementary Figure S1b. [file 41598_2020_72270_MOESM2_ESM.tiff]

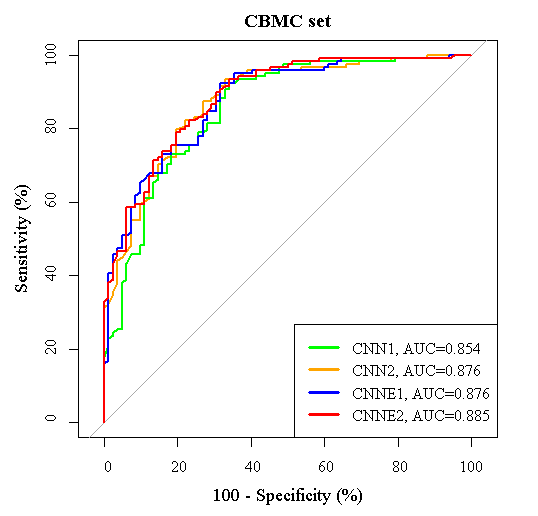

Supplement: Supplementary file 3 — Supplementary Figure S1c. [file 41598_2020_72270_MOESM3_ESM.tiff]

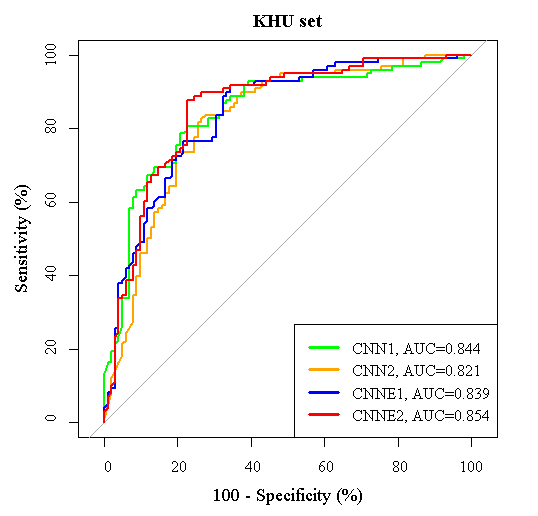

Supplement: Supplementary file 4 — Supplementary Figure S1d. [file 41598_2020_72270_MOESM4_ESM.tiff]
