# Supplementary material for: Diagnosis of thyroid nodules on ultrasonography by a deep convolutional neural network
Source: Sci Rep. 2020 Sep 17;10:15245. doi: 10.1038/s41598-020-72270-6 (PMC7498581; doi:10.1038/s41598-020-72270-6)
Supplement: Supplementary file 5 — Supplementary information. [file 41598_2020_72270_MOESM5_ESM.docx]

**Diagnosis of thyroid nodules on ultrasonography by a deep convolutional neural network**

Jieun Koh, MD^1*^, Eunjung Lee, PhD^2*^, Kyunghwa Han, PhD^3^, Eun-Kyung Kim, MD, PhD^3^, Eun Ju Son, MD, PhD^4^, Yu-Mee Sohn, MD, PhD^5^, Mirinae Seo, MD^5^, Mi-ri Kwon, MD^6^, Jung Hyun Yoon, MD, PhD^3^, Jin Hwa Lee, MD, PhD^7^, Young Mi Park, MD, PhD^8^, Sungwon Kim, MD, PhD^3^, Jung Hee Shin, MD, PhD^6†^, Jin Young Kwak, MD, PhD^3†^

Supplementary method: Ultrasound instruments used for algorithm development

One of the following 11 ultrasonography machines was used for algorithm development: HDI 3000 or 5000 (Philips-Advanced Technology Laboratories, Bothell, WA, USA) with a 7-12-MHz linear array transducer, iU22 (Philips Medical Systems, Bothell, WA, USA) with a 5-12-MHz linear array transducer, Logic 9 (GE Medical Systems, Milwaukee, WI, USA) with a 5-12-MHz linear array transducer, EPIQ5 (Philips Healthcare, Bothell, WA, USA) with a 5-12-MHz linear array transducer, SONOLINE Antares (Siemens Medical Solutions, Erlangen, Germany) with a 5-13-MHz linear array transducer, Acuson Sequoia 512 (Siemens Medical Solutions USA, Inc, Mountain View, CA, USA) with a 5-13-MHz linear array transducer, HI VISION Ascendus (Hitachi Aloka Medical, Tokyo, Japan) with a 5-13-MHz linear array transducer, EUB-7500 (Hitachi Aloka Medical, Tokyo, Japan) with a 6-14-MHz linear array transducer, Acuson Sequoia (Siemens Medical Solutions, Mountain View, CA, USA) with a 8-15-MHz linear array transducer, and EPIQ7 (Philips Medical Systems, Bothell, WA, USA) with a 4-17-MHz linear array transducer.

Supplementary Table S1. Detailed diagnostic performances of the CNNs.

|  | SH test set (n=634) | | | | SMC set (n=781) | | | | CBMC set (n=200) | | | | KUH set (n=200) | | | |
| --- | --- | --- | --- | --- | --- | --- | --- | --- | --- | --- | --- | --- | --- | --- | --- | --- |
|  | CNN1 | CNN2 | CNNE1 | CNNE2 | CNN1 | CNN2 | CNNE1 | CNNE2 | CNN1 | CNN2 | CNNE1 | CNNE2 | CNN1 | CNN2 | CNNE1 | CNNE2 |
| Sensitivity^a^ | 82.6 (79.1-85.5) | 78.0 (76.4-83.1) | 83.1 (79.7-86.1) | 82.2 (78.7-85.2) | 84.9 (81.7-87.7) | 78.6 (75.0-81.9) | 74.4 (70.5-77.9) | 77.3 (73.6-80.7) | 94.1 (88.1-97.1) | 89.0 (82.0-93.5) | 92.4 (86.0-96.0) | 94.1 (88.1-97.2) | 89.8 (82.1-94.4) | 86.7 (78.5-92.1) | 91.8 (84.5-95.9) | 91.8 (84.5-95.9) |
| Specificity^a^ | 88.4 (80.3-93.5) | 86.3 (77.9-91.9) | 91.6 (84.1-95.7) | 91.6 (84.1-95.7) | 70.8 (64.8-76.2) | 81.1 (75.7-85.5) | 87.7 (82.9-91.2) | 88.5 (83.8-91.9) | 63.4 (52.5-73.1) | 67.1 (56.2-76.4) | 64.6 (53.8-74.2) | 62.2 (51.3-72.0) | 62.8 (53.0-71.6) | 66.7 (57.0-75.1) | 61.8 (52.0-70.7) | 59.8 (50.0-68.9) |
| Accuracy^a^ | 83.4 (80.3-86.1) | 80.9 (77.7-83.8) | 84.4 (81.4-87.0) | 83.6 (80.5-86.3) | 80.5 (77.6-83.2) | 79.4 (76.4-82.1) | 78.5 (75.5-81.2) | 80.8 (77.9-83.4) | 81.5 (75.5-86.3) | 80.0 (73.9- 85.0) | 81 (75.0-85.9) | 81 (75.0-85.9) | 76 (70.0-81.4) | 76.5 (70.1-81.9) | 76.5 (70.1-81.9) | 75.5 (69.1-81.0) |
| PPV^a^ | 97.6 (95.7-98.7) | 97.1 (95.0-98.3) | 98.3 (96.5-99.1) | 98.2 (96.5-99.1) | 86.6 (83.4-89.2) | 90.2 (87.2-92.6) | 93.0 (90.2-95.1) | 93.7 (91.0-95.6) | 78.7 (71.2-84.7) | 79.6 (71.8-85.6) | 79.0 (71.4-85.0) | 78.2 (70.6-84.2) | 69.8 (61.3-77.2) | 71.4 (62.7-78.8) | 69.8 (61.3-77.1) | 68.7 (60.3-76.1) |
| NPV^a^ | 47.2 (40.0-54.5) | 43.2 (36.3-50.3) | 48.9 (41.6-56.2) | 47.5 (40.4-54.8) | 68.0 (62.0-73.4) | 63.1 (57.7-68.3) | 60.7 (55.5-65.7) | 63.8 (58.5-68.8) | 88.1 (77.1-94.2) | 80.9 (69.8-88.6) | 85.5 (74.4-92.3) | 87.9 (76.8-94.1) | 86.5 (76.7-92.6) | 84.0 (74.3-90.5) | 88.7 (79.1-94.3) | 88.4 (78.5-94.1) |
| AUC | 0.898 (0.865-0.930) | 0.911 (0.882-0.940) | 0.937 (0.914-0.959) | 0.935 (0.912-0.959) | 0.854 (0.827-0.881) | 0.872 (0.846-0.897) | 0.876 (0.852-0.900) | 0.881 (0.857-0.905) | 0.854 (0.800-0.908) | 0.876 (0.828-0.924) | 0.876 (0.828-0.924) | 0.885 (0.839-0.930) | 0.844 (0.788-0.900) | 0.821 (0.762-0.881) | 0.839 (0.783-0.894) | 0.854 (0.800-0.908) |
| F1 | 90.1 | 89.5 | 89.5 | 87.7 | 82.6 | 84.7 | 85.7 | 84.0 | 85.2 | 85.4 | 85.7 | 84.0 | 79.3 | 78.6 | 78.6 | 78.3 |

Note: ^a^To calculate the diagnostic performances of each cohort, the cut-off value of cancer probability was calculated from the SH test set according to Youden’s index (0.6 for CNN1, 0.5 for CNN2, 0.6 for CNNE1, and 0.6 for CNNE2).

Supplementary Table S2. Malignancy rates of thyroid nodules when the cancer possibility of CNNE2 was reclassified according to the ACR TI-RADS categories.

| ACR TI-RADS category | SH test | | | SMC | | | CBMC | | | KHU | | | Total | | |
| --- | --- | --- | --- | --- | --- | --- | --- | --- | --- | --- | --- | --- | --- | --- | --- |
|  | Total nodule | Malignant nodule | Malignant rate (%) | Total nodule | Malignant nodule | Malignant rate (%) | Total nodule | Malignant nodule | Malignant rate (%) | Total nodule | Malignant nodule | Malignant rate (%) | Total nodule | Malignant nodule | Malignant rate (%) |
| 2  (< 2 %) | 3 | 0 | 0.0 | 0 | 0 |  | 0 | 0 |  | 0 | 0 |  | 3 | 0 | 0.0 |
| 3  (2-5 %) | 6 | 1 | 16.7 | 0 | 0 |  | 1 | 0 | 0.0 | 2 | 0 | 0.0 | 9 | 1 | 11.1 |
| 4  (5-20 %) | 43 | 13 | 30.2 | 89 | 17 | 19.1 | 19 | 1 | 5.3 | 19 | 1 | 5.3 | 170 | 32 | 18.8 |
| 5  (≥ 20 %) | 582 | 525 | 90.2 | 692 | 521 | 75.3 | 180 | 117 | 65.0 | 179 | 97 | 54.2 | 1633 | 1260 | 77.2 |
|  | 634 | 539 | 85.0 | 781 | 538 | 68.9 | 200 | 118 | 59.0 | 200 | 98 | 49.0 | 1815 | 1293 | 71.2 |

Note: Numbers in parentheses are recommended risks of malignancy according to the ACR TI-RADS

Supplementary Figure S1. ROC curves of the four CNNs for differentiating malignant thyroid nodules. CNNs demonstrated high AUC to differentiate malignant thyroid nodules which were A. 0.898-0.937 for the SH test set, B. 0.854-0.881 for the SMC set, C. 0.854-0.885 for the CBMC set, D. 0.821-0.854 for the KUH set. For the SH test set, CNNE1 and CNNE2 showed higher AUC compared to CNN1 or CNN2. (CNN1: green, CNN2: orange, CNNE1: blue, CNNE2: red).
